# A bibliometric analysis of research on the application of just-in-time adaptive interventions in mental health

**DOI:** 10.1097/MD.0000000000050540

**Published:** 2026-09-04

**Authors:** Lu Luo, Aimin Wang, Jianhui Zhou, Pan Su, Lichun Tang, Pingfang Liu, Wenjun Xie, Congling Li

**Affiliations:** a Hunan Provincial Maternal and Child Health Care Hospital, Changsha, Hunan, China; b Emergency Department, Xiangya Hospital, Central South University, Changsha, Hunan, China; c Teaching and Research Section of Clinical Nursing, Xiangya Hospital, Central South University, Changsha, Hunan, China; d Emergency Department, Hunan University of Medicine General Hospital, Huaihua, Hunan, China; e Nursing Department, Hunan University of Medicine General Hospital, Huaihua, Hunan, China.

**Keywords:** bibliometric analysis, ecological momentary assessment, just-in-time adaptive intervention, mental health, micro-randomized trials

## Abstract

**Objective:**

To systematically review the current status, hotspots, knowledge structure, and evolutionary trends of research on just-in-time adaptive interventions (JITAIs) in the field of mental health using bibliometric methods, providing references for future research and practice.

**Methods:**

Literature related to JITAI and mental health was retrieved from the Web of Science Core Collection database, covering the period from its inception to January 6, 2026. CiteSpace software was used to analyze publication trends, collaboration networks, co-citation clustering, and keywords of the 451 ultimately included articles, with the results visualized.

**Results:**

The annual publication volume has shown explosive growth since 2021, reaching a peak in 2025 (117 articles). The United States ranked 1st with 304 publications and a centrality of 0.59. The University of Michigan had the highest number of publications (51 articles), while the University of Southern California had the highest centrality (0.14) in the institutional collaboration network. Michael S. Businelle had the highest publication count (20 articles). Keyword clustering identified 14 core themes, including ecological momentary assessment, mental health, and weight management. The research trajectory has evolved from methodological innovations towards clinical applications and differentiated interventions for specific populations. Burst keyword analysis indicates that current research frontiers focus on ecological momentary interventions, micro-randomized trials, and data reliability validation. Combining clustering and burst keywords, research hotspots can be summarized into 3 main directions: methodological innovations centered on micro-randomized trials and reinforcement learning; clinical applications targeting emotional and behavioral issues; and differentiated interventions for specific populations and contexts.

**Conclusion:**

The application of JITAIs in mental health is developing rapidly and has become a hot topic in interdisciplinary research. However, research capacity is unevenly distributed, and international collaboration needs strengthening. Future efforts should focus on cross-regional and interdisciplinary collaboration, deepen validation of intervention effectiveness, ensure data reliability, and establish ethical guidelines to promote the field towards precise, scalable, and responsible applications.

## 1. Introduction

With the rapid advancement of mobile sensing technologies, just-in-time adaptive interventions (JITAIs), as an emerging intervention paradigm, offer revolutionary prospects for the field of mental health. JITAIs aim to use mobile devices and sensors to capture an individual’s dynamically changing internal and contextual states in real-time, thereby striving to “deliver the right type and dose of support at the right time.”^[1]^ Their core advantage lies in their ability to transcend the limitations of traditional interventions regarding accessibility, timeliness, and personalization. This provides a highly promising and precise tool for addressing a range of mental health issues, from substance use disorders to emotional distress.

In the intervention of addictive behaviors, the value of JITAIs is particularly prominent. Since “timing is crucial for relapse prevention,” mobile technologies capable of providing “anytime, anywhere” emotional and instrumental support can significantly extend continuous care for patients.^[2]^Research indicates that JITAIs targeting substances like alcohol and tobacco, utilizing ecological momentary assessment (EMA) and static decision rules, can offer tailored support in response to micro-level fluctuations in emotion or craving.^[3]^ For eating disorders, particularly binge-eating disorder and bulimia nervosa, the efficacy of cognitive behavioral therapy(CBT) relies on the acquisition and application of therapeutic skills. JITAI systems (e.g., CBT+) are designed to “provide real-time intervention at algorithmically identified opportunities for skill practice,” serving as an effective adjunct to face-to-face therapy to improve skill utilization and symptom outcomes.^[4]^ JITAIs also demonstrate application potential in mood disorders. For instance, Mello, a fully automated JITAI targeting repetitive negative thinking, can “provide personalized, contextually sensitive support in real time when it is needed,” with preliminary trials showing its effectiveness in alleviating depressive and anxiety symptoms in youth.^[5]^Furthermore, JITAIs offer a new approach for suicide prevention. By utilizing smartphones and wearable devices to “provide the right support when needed by adapting to changes in internal states and external environments,” they hold promise for better matching the dynamic and heterogeneous nature of suicidal ideation and behavior.^[6]^

To scientifically construct and optimize JITAIs, novel experimental designs such as micro-randomized trials (MRTs) have emerged. MRTs, by randomizing individuals at high-frequency time points, can “test whether an intervention component exerts its intended effect, when it works, for whom it works, and what factors moderate the intervention effect,” thereby providing causal evidence for creating more effective JITAIs^[7]^ However, the development of this field faces several challenges. Existing health behavior theories are “insufficient for guiding the development of interactive, adaptive interventions.”^[8]^ Many commercially available mental health apps “have not yet leveraged JITAI mechanisms to tailor content based on context, state, or person.”^[9]^ Research also indicates that evidence for the effectiveness of JITAIs in areas such as substance use remains insufficient, and many studies lack detailed reporting on JITAI infrastructure, content, development costs, and data security.^[3]^

In summary, the application of JITAI in mental health represents a cross-disciplinary, rapidly evolving, and promising frontier field.^[10,11]^ Its research encompasses a broad range of topics, from theoretical frameworks and experimental methods to applications for specific disorders. While the current literature shows a rapid growth trend, there is a lack of systematic organization regarding research themes, methodologies, and application outcomes. Bibliometrics, as a big-data-based quantitative analysis method, can intuitively reveal the research hotspots, knowledge foundation, evolutionary trajectory, and development frontiers of a field by analyzing publication trends, collaboration networks, keyword co-occurrence, and literature co-citation. Therefore, this study aims to employ bibliometric methods to visually analyze the current state of research on the application of JITAIs in mental health, in order to clarify the knowledge structure of this field, identify core themes and evolutionary paths, and provide references for future research and practice directions.

## 2. Materials and methods

### 2.1. Data sources

**Figure 1.**
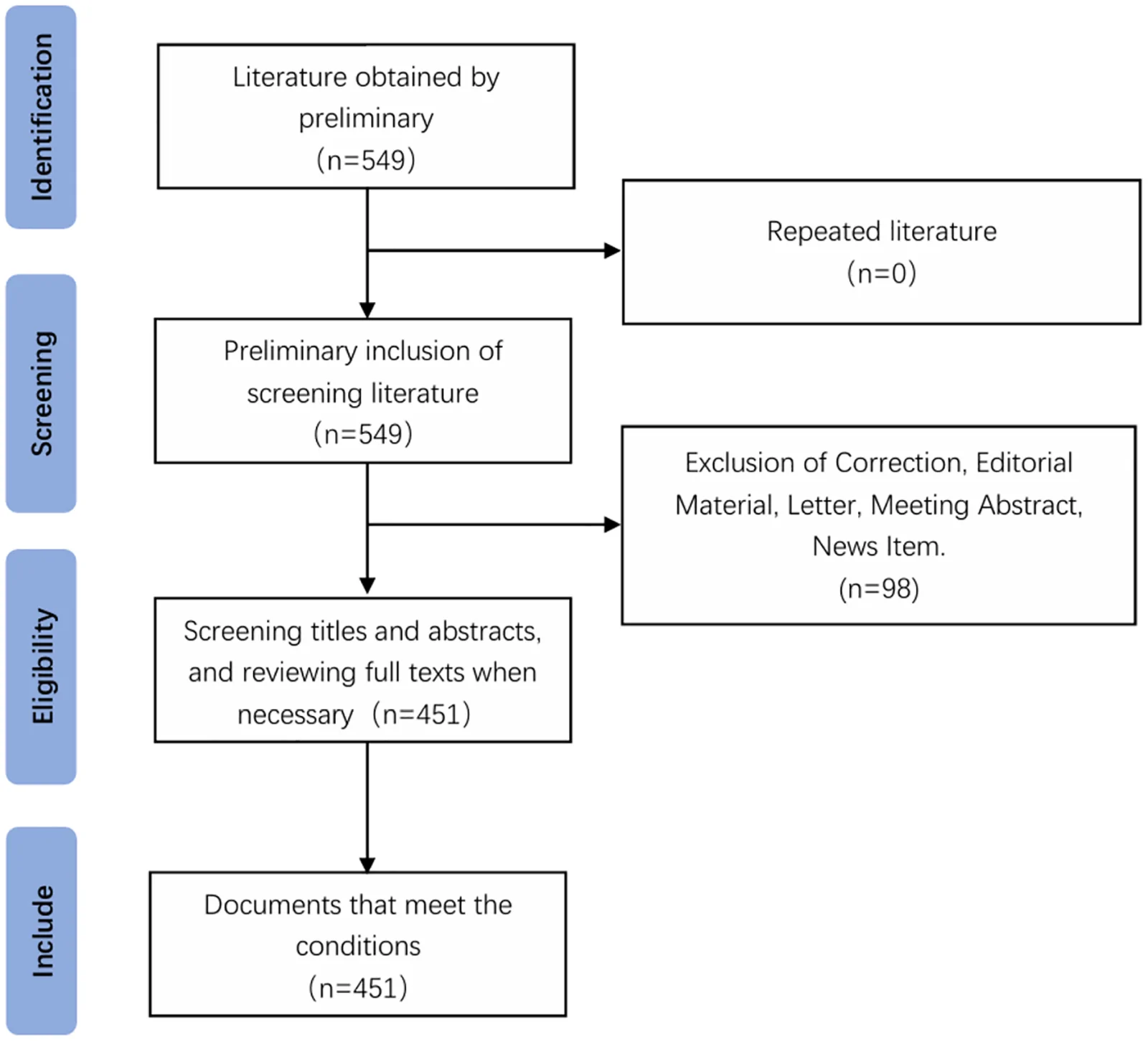
Retrieval flow chart.

A search strategy was constructed using the advanced search function within the Web of Science (WOS) database. The search timeframe was set from the database’s inception to January 6, 2026. The search strategy for the WOS Core Collection was as follows: TS = ((“just-in-time” OR “JITAI” OR “just in time”)) AND TS = ((“adaptive intervention*” OR “adaptive treatment” OR “dynamic treatment regimen*” OR “personalized intervention*” OR “tailored intervention*” OR “ecological momentary intervention*” OR “EMI”)) AND TS = ((“mental health” OR “mental disorder*” OR “psych*” OR “depress*” OR “anxi*” OR “stress” OR “substance use” OR “alcohol” OR “smoking cessation” OR “well-being”)). While databases such as PubMed and Scopus offer broader coverage of biomedical literature, this study deliberately restricted data retrieval to the WOS Core Collection. This decision was based on WOS’s provision of standardized, high-quality citation data (including cited references), which is essential for the reliable computation of co-citation analysis and betweenness centrality in CiteSpace. A single-source strategy ensures data consistency and avoids the technical complexities and potential biases introduced by merging deduplicated records from multiple platforms.The literature types were filtered to include only articles and reviews. The initial search yielded 549 records. By reviewing titles and abstracts, and reading the full text when necessary, 55 proceeding papers, 35 meeting abstracts, 8 early access publications, 8 editorial materials, 1 book chapter, and 1 correction were excluded, resulting in a final inclusion of 451 articles for analysis. Literature screening was independently conducted by 2 researchers. Obvious irrelevant records were excluded by reading titles and abstracts, and the remaining literatures were reviewed in full-text to determine the final included studies. In case of disagreements during the screening process, they were resolved through discussion or consultation with a 3rd researcher to ensure a high degree of consistency in the application of inclusion criteria. The data from the included literature originated from 236 countries/regions, involved 1717 institutions, and 2204 authors. The screening process is illustrated in Figure 1.

### 2.2. Research methods

This study employed CiteSpace 6.3.R1 software to conduct a visual analysis of the included 451 articles. The analysis was set with a time span from the database’s inception to January 6, 2026, using a 1-year time slice. The node selection strategy was set to the top 50 high-frequency nodes per slice (top N = 50), with the pruning method set to Pathfinder. Separate collaboration networks for countries, institutions, and authors were constructed, along with a keyword co-occurrence network. Research themes were identified through cluster analysis, and research frontiers were revealed through burst detection.^[12–14]^ Clusters were labeled using the log-likelihood ratio algorithm, and the *Q* value of 0.6525 (>0.3) indicates a significant cluster structure, while the *S* value of 0.8392 (>0.5) suggests high cluster homogeneity. In the knowledge maps generated by CiteSpace, N represents the number of network nodes, *E* represents the number of links, and density indicates network density. Centrality represents the importance of a node within the map. The quality of the map can be evaluated using Modularity *Q (Q* value) and Silhouette (*S* value), where *Q* > 0.3 indicates a significant cluster structure, and *S* > 0.5 suggests that the clusters are reasonable and possess high homogeneity. Centrality reflects the influence and connectivity of a node within the network of the knowledge map. The knowledge map uses citation rings to represent the influence of corresponding nodes, while the frequency and thickness of the lines reflect the co-occurrence relationships between nodes.

## 3. Results

### 3.1. Temporal distribution of publications

**Figure 2.**
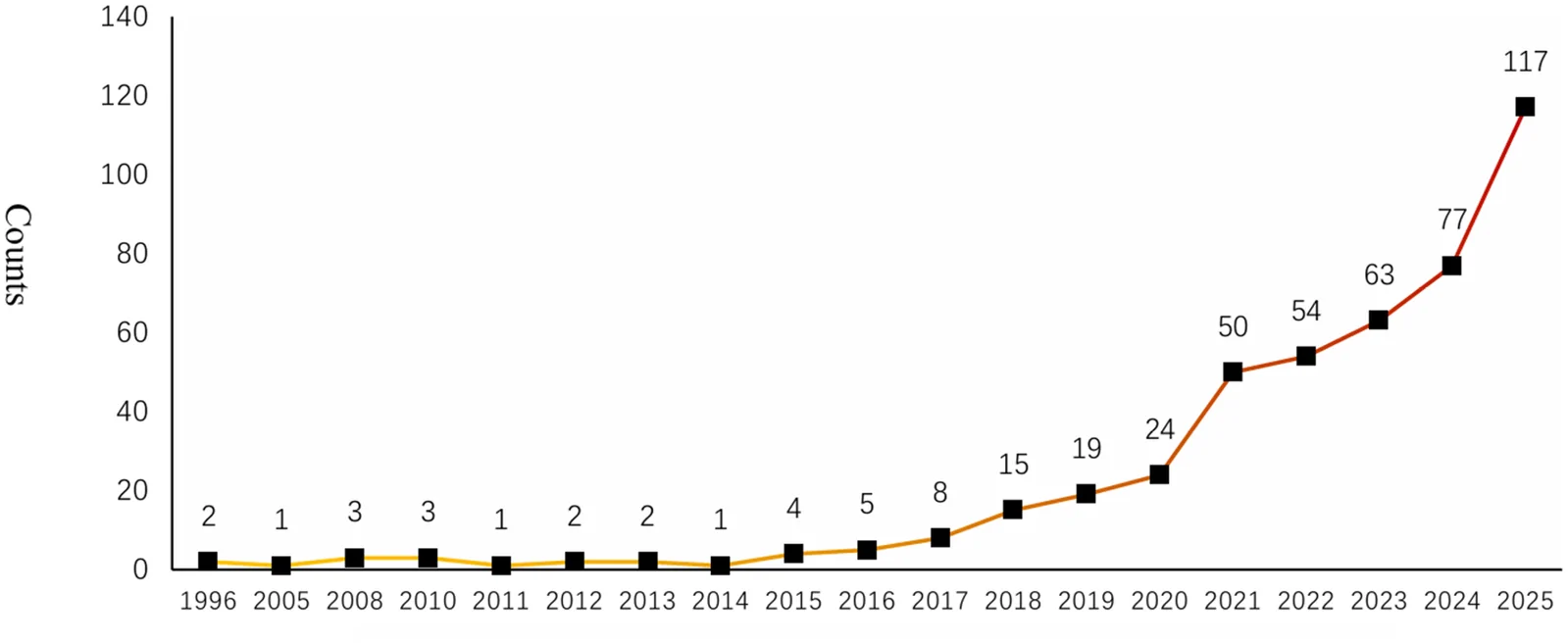
Analysis of annual publications.

Figure 2 illustrates the annual publication trend of research on the application of JITAIs in mental health. Analysis of the data on the number of publications over the years reveals a clear developmental trajectory for research in this field. Overall, the volume of literature on this topic has increased annually over time, with particularly notable growth observed in the most recent 5 years. This trend reflects the growing attention from both academia and practice, likely driven by factors such as the proliferation of digital mental health technologies, increased demand for personalized interventions, and advancements in related interdisciplinary research methodologies. In terms of developmental stages, the low annual publication output in the early years indicates an initial exploratory phase. This was followed by a period of steady accumulation, during which research topics gradually focused on intervention mechanisms and outcome evaluation. The rapid growth in recent years likely corresponds to the integration of real-time monitoring technologies, artificial intelligence algorithms, and mobile health applications, fostering deeper investigation into the accessibility, adaptability, and effectiveness of just-in-time psychological interventions. This developmental trend also suggests that future research could further address issues such as the clinical translation of intervention technologies, verification of long-term effects, cultural adaptation, and ethical guidelines. These efforts would promote a more systematic and robust development of the field, providing a solid scientific foundation for the precision and timeliness of mental health services.

### 3.2. Country distribution

**Figure 3.**
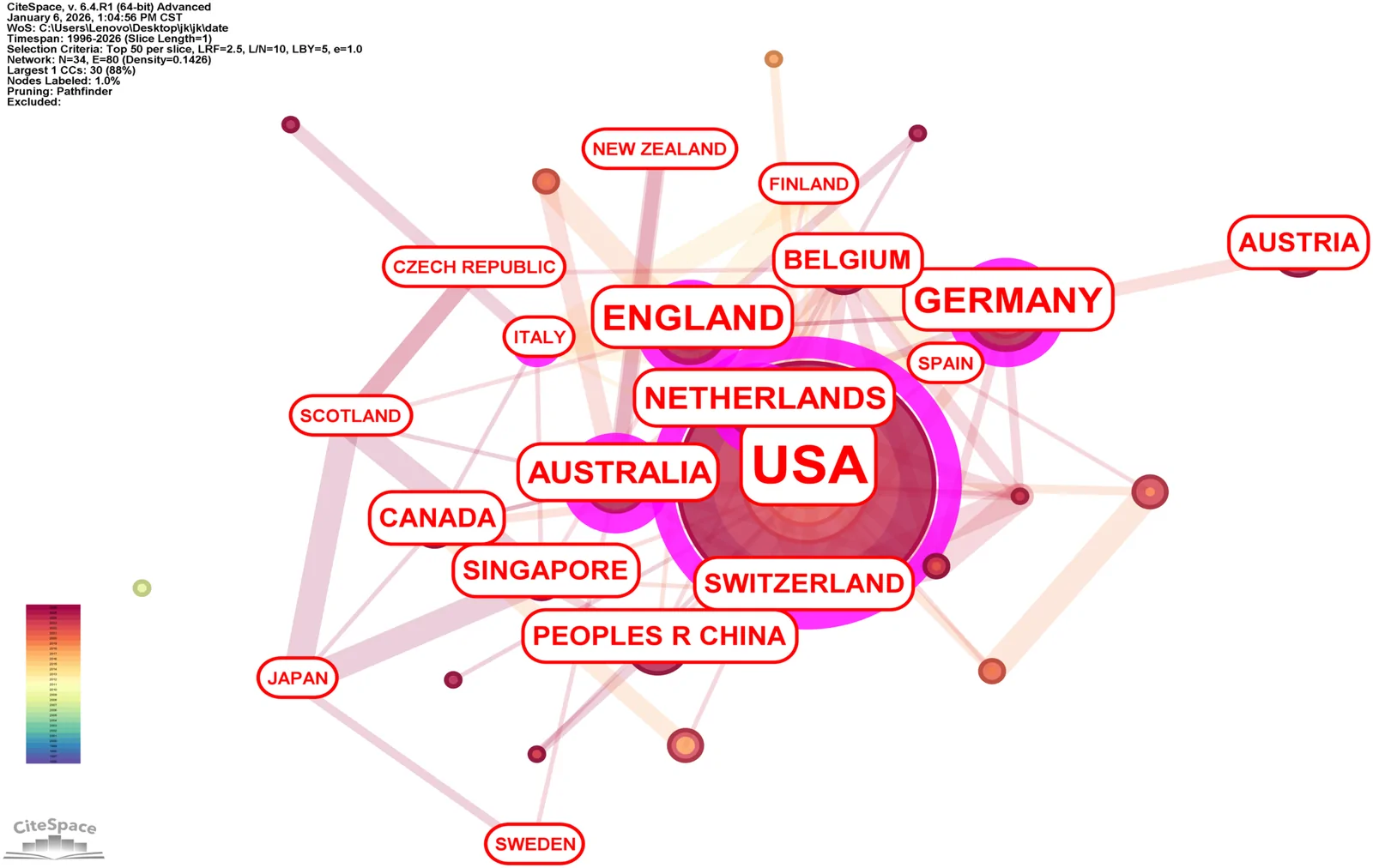
Collaborating countries.

A knowledge map of country collaboration networks was generated using the following parameters: node type as country, selection of the top 50 high-frequency nodes per slice (top N = 50), a time span from 1996 to 2025, and the Pathfinder pruning method. The result is shown in Figure 3. Figure 3 indicates close collaboration among countries. The USA has the highest number of publications (304), followed by Germany (40) and England (36), indicating these countries are the primary contributors to current research in this field. In terms of centrality, the USA (0.59) ranks 1st, followed by Australia (0.31) and England (0.16). The country publication counts and centrality distribution results are presented in Table 1.

**Table 1:** Countries distribution.

Country	Count	Country	Centrality
USA	302	USA	0.6
Germany	40	Australia	0.34
England	36	England	0.18
Australia	30	Netherlands	0.14
Netherlands	23	Canada	0.14
Canada	20	Germany	0.13
Switzerland	17	Italy	0.12
Belgium	16	Belgium	0.09
Peoples R China	16	Switzerland	0.05
Austria	15	Peoples R China	0.04

### 3.3. Institutional distribution

**Figure 4.**
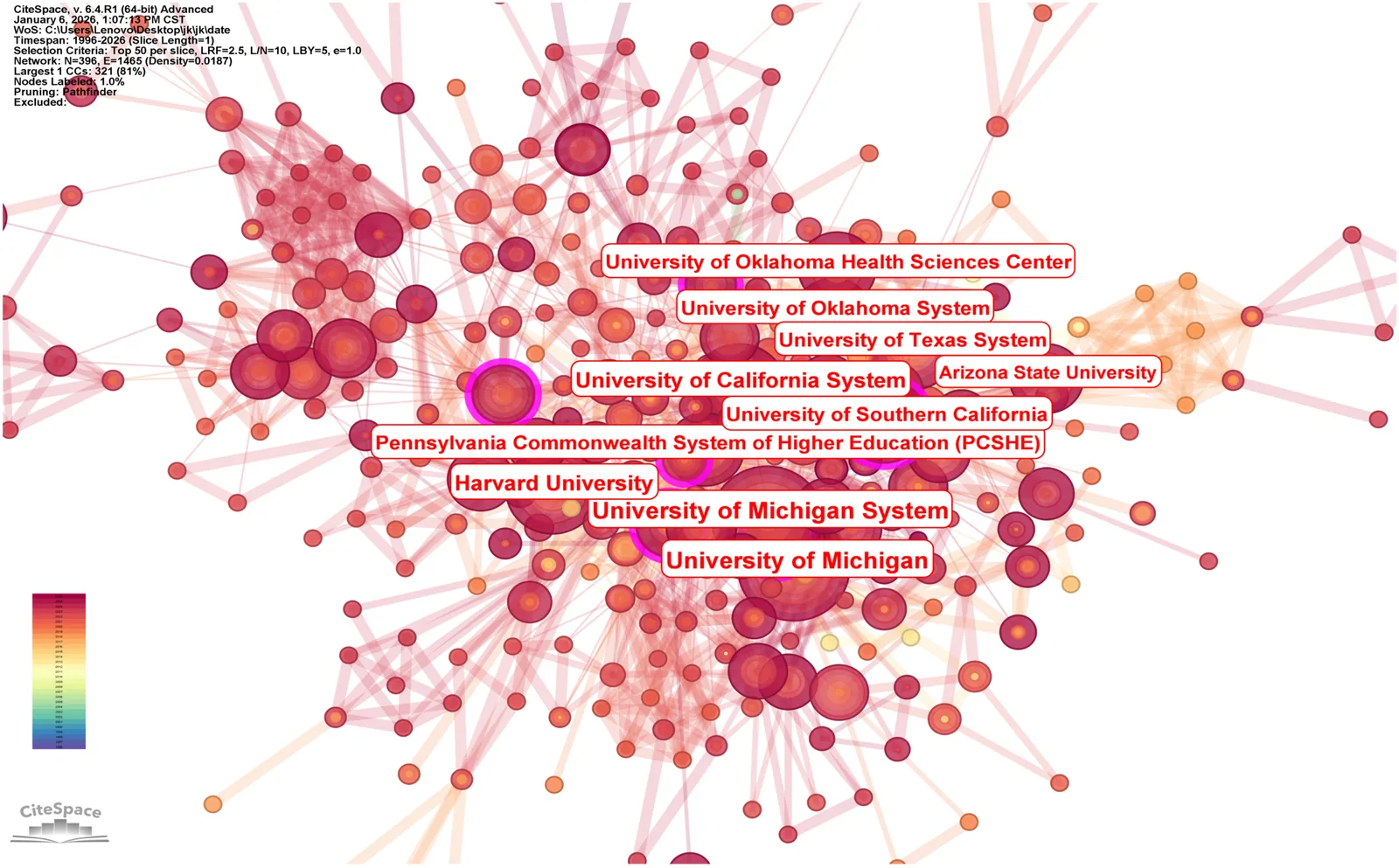
Knowledge map of institutional cooperation.

A knowledge map of institutional collaboration networks was generated using the following strategy: selecting institution as the node type, top N = 50, a time span from 1996 to 2025, and the Pathfinder pruning method. The result is shown in Figure 4. Figure 4 shows that collaboration among institutions is relatively close and frequent. The institution with the highest publication count is the University of Michigan (51 articles), followed by the University of California System (43 articles) and Harvard University (34 articles). Furthermore, the institution with the highest centrality is the University of Southern California (0.14), followed by the University of California Los Angeles (0.13), Northeastern University (0.13), and Brown University (0.12). The institutional publication counts and centrality distribution results are presented in Table 2.

**Table 2:** Institutions distribution.

Institution	Count	Institution	Centrality
University of Michigan	50	University of Southern California	0.15
University of Michigan System	50	Northeastern University	0.15
University of California System	43	Brown University	0.11
Harvard University	34	Harvard Medical School	0.09
Pennsylvania Commonwealth System of Higher Education (PCSHE)	31	University of London	0.08
University of Oklahoma System	29	National Institutes of Health (NIH) – USA	0.08
University of Oklahoma Health Sciences Center	29	University of California San Diego	0.08
University of Texas System	25	University of California Los Angeles	0.08
University of Southern California	24	University College London	0.08
Arizona State University	20	University of Amsterdam	0.08

### 3.4. Author distribution

**Figure 5.**
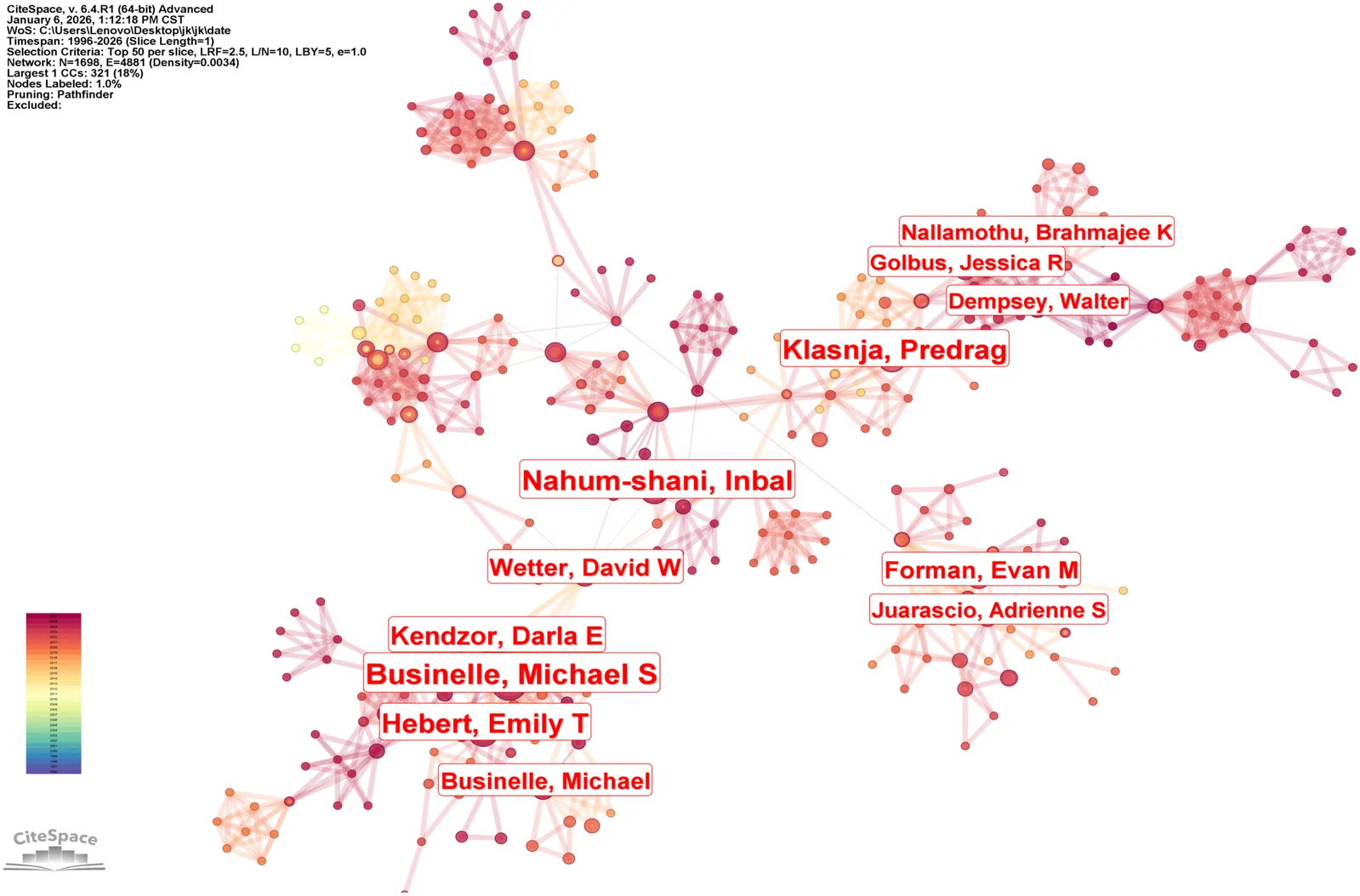
Knowledge map of author cooperation.

An author collaboration network knowledge map was generated using the following strategy: node type set to author, top N = 50, time span from 2000 to 2025, and Pathfinder pruning. The result is shown in Figure 5. Figure 5 indicates that collaboration among authors is relatively loose. Businelle, Michael S has the highest number of publications (20), followed by Nahum-shani, Inbal (17), Hardeman W Klasnja (15), and Hebert, Emily T (15), indicating these authors are the primary contributors to current research in this field. In terms of centrality, Wetter, David W (0.03), Qian, Tianchen (0.03), and Perski, Olga (0.03) have the highest centrality, followed by Klasnja, Predrag (0.02), Goldstein, Stephanie P (0.02), among others. The author publication counts and centrality distribution results are presented in Table 3.

**Table 3:** Authors distribution.

Author	Count	Author	Centrality
Businelle, Michael S	20	Wetter, David W	0.03
Nahum-shani, Inbal	17	Qian, Tianchen	0.03
Klasnja, Predrag	15	Perski, Olga	0.03
Hebert, Emily T	15	Klasnja, Predrag	0.02
Kendzor, Darla E	13	Goldstein, Stephanie P	0.02
Wetter, David W	10	Murphy, Susan A	0.02
Forman, Evan M	10	Brown, Jamie	0.02
Businelle, Michael	9	Collins, Linda M	0.02
Dempsey, Walter	8	Businelle, Michael S	0.01
Nallamothu, Brahmajee K	8	Nahum-shani, Inbal	0.01

### 3.5. Analysis of research hotspots

**Figure 6.**
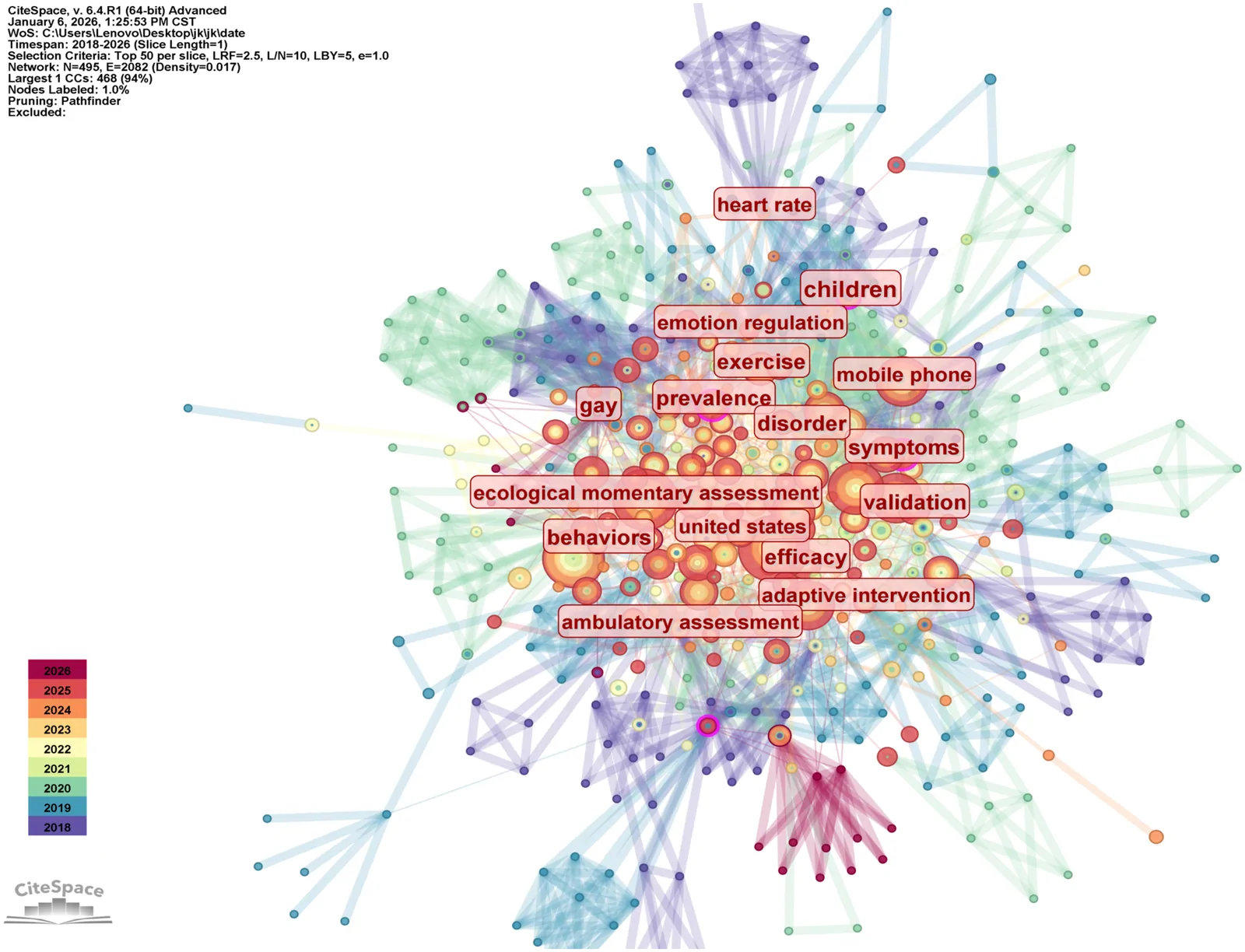
Knowledge map of keywords.

A keyword knowledge map was generated using the following strategy: node type set to keyword, top N = 50, time span from 2018 to 2026, and Pathfinder pruning. The map contains 495 nodes and 2082 links, with a density of 0.017. The result is shown in Figure 6.

The top 10 keywords by frequency are: just-in-time adaptive intervention, ecological momentary assessment, physical activity, behavior, mobile health, mobile phone, health, smoking cessation, mental health, and meta-analysis. The top 10 keywords by centrality are: smoking cessation, quality, ecological momentary assessment, behavior change, ecological momentary intervention, symptoms, strategy, efficacy, prevalence, and drinking. Keyword information is detailed in Table 4.

**Table 4:** Keywords distribution.

Keyword	Count	Keyword	Centrality
just-in-time adaptive intervention	84	smoking cessation	0.11
ecological momentary assessment	83	quality	0.11
behavior	66	ecological momentary assessment	0.08
physical activity	63	behavior change	0.08
mobile health	54	ecological momentary intervention	0.08
health	51	symptoms	0.08
mobile phone	51	strategy	0.08
mental health	40	efficacy	0.07
smoking cessation	40	prevalence	0.07
meta-analysis	33	drinking	0.07
technology	28	children	0.07
adults	27	ambulatory assessment	0.07
just-in-time adaptive interventions	27	quality	0.11
stress	26	symptoms	0.11
digital health	26	prevalence	0.08
models	26	children	0.08
interventions	25	ecological momentary assessment	0.08
validation	24	efficacy	0.08
machine learning	22	validation	0.08
efficacy	22	adaptive intervention	0.07

**Figure 7.**
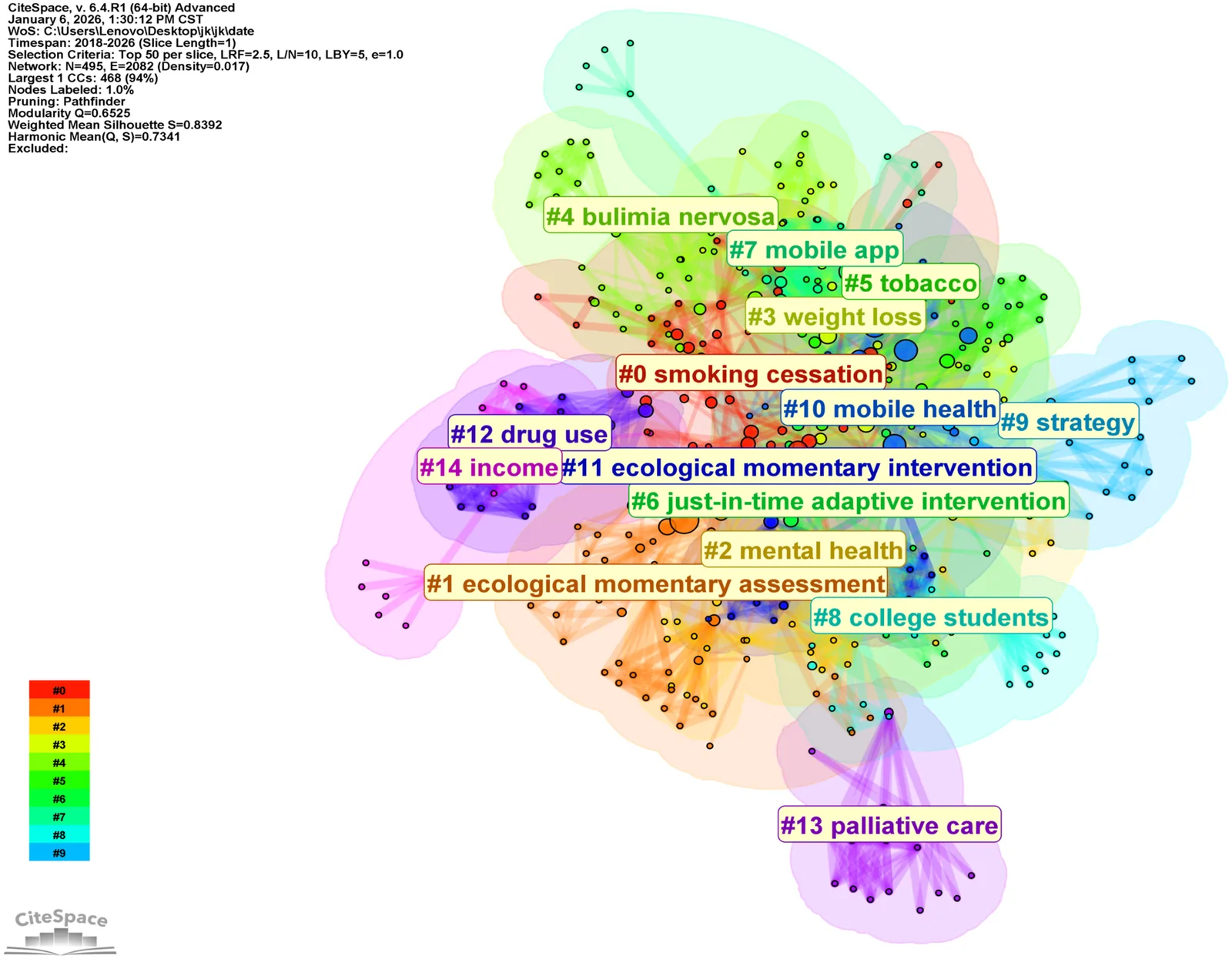
Knowledge map of keyword clustering.

Based on Figure 6, a cluster analysis of keywords was conducted. The results are presented in Figure 7. The cluster analysis map has a modularity *Q* of 0.6525 and a weighted average silhouette coefficient *S* of 0.8392, indicating good clustering effectiveness. In Figure 7, clusters are labeled as # + number + name, representing the cluster sequence number and name. A total of 14 significant clusters were identified. The cluster information is detailed in Table 5, with the cluster names being: ecological momentary assessment, mental health, weight loss, bulimia nervosa, tobacco, just-in-time adaptive intervention, mobile app, college students, strategy, mobile health, ecological momentary intervention, drug use, palliative care, and income.

**Table 5:** Keywords clustering information.

Cluster ID	Size	Silhouette	Mean (year)	Top terms (log-likelihood ratio, *P* level)
0	66	0.62	2020	Smoking cessation (9.32, .005); psychological inflexibility (7.23, .01); micro-interventions (7.23, .01); personalized medicine (7.23, .01); qualitative (7.23, .01)
1	48	0.773	2020	Ecological momentary assessment (EMA) (12.86, .001); heart rate variability (12.41, .001); just-in-time adaptive intervention (JITAI) (8.84, .005); machine learning (8.74, .005); alcohol use disorder (8.61, .005)
2	38	0.895	2019	Mental health (18.6, 1.0E−4); just-in-time interventions (18.19, 1.0E−4); behavioral health (7.81, .01); intensive longitudinal data (7.81, .01); adults (7.81, .01)
3	37	0.768	2019	Weight loss (15.7, 1.0E−4); digital mental health (11.5, .001); diet (8.04, .005); lapses (7.56, .01); adaptive intervention (6.47, .05)
4	35	0.931	2019	Bulimia nervosa (24.95, 1.0E−4); binge-eating disorder (14.89, .001); emotion regulation (14.21, .001); technology (11.21, .001); major depressive disorder (7.43, .01)
5	35	0.898	2019	Tobacco (10.5, .005); just-in-time adaptive interventions (6.51, .05); just-in-time adaptive intervention (6.11, .05); smoking cessation (5.42, .05); health care access (4.87, .05)
6	33	0.771	2021	just-in-time adaptive intervention (13.85, .001); continuous glucose monitoring (11.89, .001); mobile health (8.69, .005); simultaneous use (7.14, .01); intentions (7.14, .01)
7	26	0.958	2019	Mobile app (17.37, 1.0E−4); digital intervention (12.64, .001); telemedicine (12.24, .001); family (9.74, .005); vaping (9.74, .005)
8	25	0.972	2022	College students (14.77, .001); substance use disorder recovery (7.37, .01); problematic pornography use (7.37, .01); behavioral adaptation (7.37, .01); crossover trial (7.37, .01)
9	24	0.884	2019	Strategy (8.99, .005); cardiorespiratory fitness (7.2, .01); psychological theory (7.2, .01); psychology social (7.2, .01); acute myocardial infarction (7.2, .01)
10	24	0.841	2019	Just-in-time adaptive intervention (25.06, 1.0E−4); mobile health (16.93, 1.0E−4); just-in-time adaptive interventions (10.41, .005); chronic disease (7.09, .01); classification (7.09, .01)
11	22	0.953	2019	Ecological momentary intervention (13.89, .001); affect (11.23, .001); ambulatory assessment (8.77, .005); ecological momentary assessment (7.26, .01); mental illnesses (6.82, .01)
12	21	0.947	2018	Drug use (8.16, .005); ecological momentary assessments (8.04, .005); youth experiencing homelessness (7.05, .01); pandemic (6.77, .01); electronic momentary assessment (6.77, .01)
13	18	0.962	2018	Palliative care (11.73, .001); rct (11.73, .001); perceptions (11.73, .001); school-based prevention (11.73, .001); supportive care (11.73, .001)
14	16	0.877	2019	Income (7.52, .01); low socioeconomic status (7.52, .01); perceived discrimination (7.52, .01); transdermal alcohol sensor (7.52, .01); hispanic/latino (7.52, .01)

**Figure 8.**
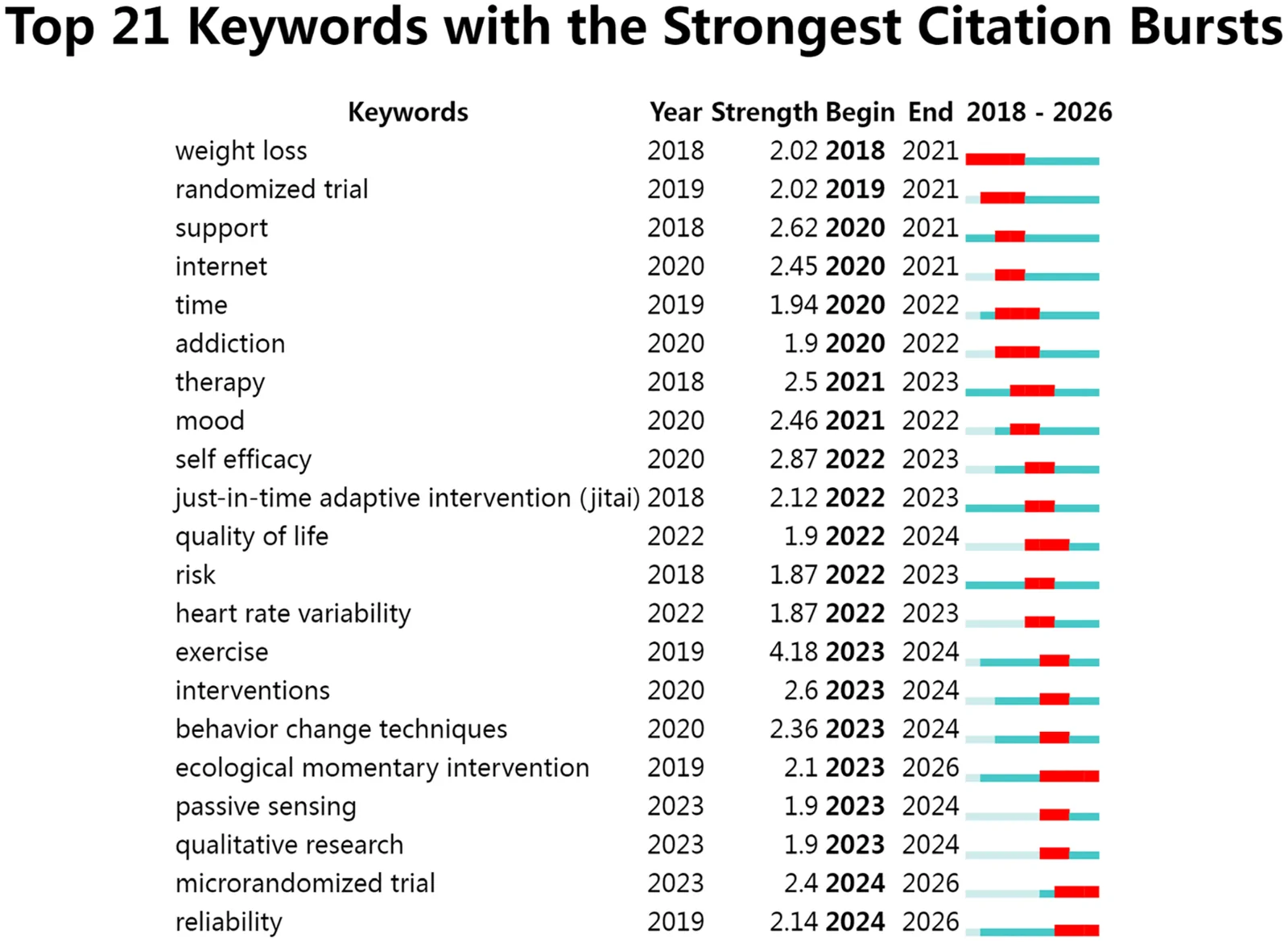
Burst keywords.

To further explore the research frontiers in this field, a burst keyword analysis was performed based on Figure 6. The results are shown in Figure 8, revealing 16 burst keywords: heart rate, children, emotion regulation, exercise, mobile phone, prevalence, disorder, symptoms, gay, ecological momentary assessment, validation, behaviors, United States, efficacy, adaptive intervention, and ambulatory assessment.

## 4. Discussion

### 4.1. Distribution of research strength

The volume of literature in this research field exhibits significant phased growth over time. In the early period (1996–2014), publication output was sparse and unstable, with annual publications consistently below 3 articles, indicating that the field was in a nascent stage of conceptual development and technical preparation. Starting from 2015, the number of publications began to increase steadily (4 articles in 2015), marking entry into a phase of stable growth. The annual publication output reveals a 3-stage evolutionary trajectory. The initial phase (1996–2014) was marked by sporadic output (<3 articles/yr). A steady growth phase began in 2015 (4 articles), followed by an explosive phase after 2021, peaking at 117 articles in 2025. This quantitative surge, visualized in Figure 2, indicates that the field has transitioned from a peripheral topic to a frontline research area within the past 5 years, coinciding with growing interest in artificial intelligence and big data technologies and increased impetus from multidisciplinary research. Overall, the field has rapidly evolved from a peripheral topic into one of the most active frontiers in current mental health research and is currently in a phase of rapid advancement characterized by theoretical deepening and technological application. This rapid evolution aligns with broader trends identified in recent bibliometric studies, which have mapped research landscapes in mental health, schizophrenia genetics, behavioral addictions, and artificial intelligence applications, further validating the importance of quantitative synthesis in this domain.^[15–17]^

### 4.2. National distribution

According to the provided data on country/region distribution, international collaboration and research influence in this field exhibit a centralized pattern dominated by a few developed nations. The United States holds a commanding lead in both total publication volume and centrality, establishing its core leadership position and absolute influence in this research domain. This dominance can be attributed to several factors, including substantial research funding from the National Institutes of Health for mobile health and digital intervention studies, advanced technological infrastructure (e.g., widespread smartphone penetration and wearable device adoption), and a strong interdisciplinary research culture that bridges computer science, behavioral medicine, and psychiatry.Countries such as Australia, England, Germany, and the Netherlands rank at the top in both publication volume and centrality, forming a solid core research bloc spanning Europe, America, and Oceania. Notably, for some countries like Australia, their centrality ranking is significantly higher than their publication volume ranking, suggesting they play a crucial intermediary or bridging role within the academic collaboration network, exerting influence beyond their mere output quantity. In contrast, countries like China (Peoples R China) ranked 9th in publication volume but had a centrality of only 0.05 (data not shown in Table 1, but can be added). Examination of the collaboration network shows that China’s co-authorship links are mainly with the USA and Australia, with few connections to European centers such as Germany or the Netherlands. This suggests its collaborative reach is narrower than that of top-centrality countries. Overall, the current distribution of research strength is highly imbalanced. Future progress requires broader global cooperation to foster knowledge dissemination and innovation. Notably, the underrepresentation of low- and middle-income countries in this research landscape reflects broader global disparities in research funding, digital infrastructure, and access to mobile health technologies, which may limit the generalizability of current JITAI evidence to diverse populations and settings.

### 4.3. Institutional distribution

The University of Michigan, as the most prolific institution, has demonstrated broad influence, with its research frequently cited in the development of MRT methodology and its application across diverse health behaviors.^[6,7,18–21]^ Its work also extends to several cutting-edge methodological areas.^[22–25]^The institution with the 2nd-highest publication output, the University of California System, focuses its JITAI research on harm reduction interventions and systematic reviews for substance use disorders^[3,26]^; the development of real-time monitoring and personalized support strategies for mental health issues^[27,28]^; methodological innovations in MRT design and behavioral prediction algorithms,^[29–31];^ and the development and feasibility assessment of tailored interventions for high-risk populations such as homeless youth and children with cerebral palsy.^[32–35]^The institution with the 3rd-highest publication output, Harvard University, focuses its research in this field on the development and methodological validation of mobile health JITAI strategies for addictive behaviors (such as alcohol and tobacco use)^[19,36,37]^; the construction and pilot evaluation of suicide risk warning systems and personalized intervention models^[38–40]^; the optimization of sensor- and EMA-based interventions for stress management and smoking relapse prevention^[41–43]^; and the design and feasibility testing of personalized adaptive digital therapies for chronic diseases (such as cancer pain and diabetes).^[44–46]^ These institutions share common characteristics, including dedicated research centers for digital health (e.g., the University of Michigan’s mHealth Initiative), consistent funding from federal agencies (e.g., National Institutes of Health’s Science of Behavior Change program), and established interdisciplinary teams that enable large-scale, longitudinal JITAI trials.

The University of Southern California, which has the highest centrality, plays a key bridging role in the collaboration network. Its research primarily contributes to the theoretical, design, and algorithmic foundations of JITAIs.^[26,47–53]^ The institution with the 2nd-highest centrality, the University of California Los Angeles, focuses its research on the development and feasibility validation of JITAI systems for respiratory illnesses in children with cerebral palsy,^[33–35]^while also working to build and optimize general methodological frameworks and their decision rules for JITAIs in areas such as mental health.^[27,54]^ The institution with the 3rd-highest centrality, Northeastern University, focuses its research on the combined application of wearable biosensing and behavioral prediction models, aiming to warn of aggressive behavior or predict suicide risk using physiological and movement data.^[40,55]^ It also emphasizes the development of mobile health-based JITAIs to enhance physical activity levels in special populations such as those with spinal cord injuries.^[20,56–58]^ Furthermore, it is dedicated to constructing dynamic behavioral computational models and system identification methods to support health behavior theory development and personalized intervention design,^[58,59]^ and to exploring strategies for real-time prediction and intervention of behaviors like substance use based on EMA and deep learning.^[60,61]^

### 4.4. Author distribution

The research of Michael S. Businelle focuses on developing and evaluating smartphone-based JITAI technologies for substance use disorders.^[62–64]^ His work places particular emphasis on applying JITAIs to disadvantaged and underserved populations, including individuals experiencing homelessness, low-income smokers, Black communities, and those with co-occurring anxiety or human immunodeficiency virus, aiming to reduce health disparities.^[50,65–67]^ His work integrates EMA and machine learning (ML) to predict relapse risk and inform real-time personalization.^[68–70]^ Building on this, his team systematically conducts a complete research cycle from intervention prototype development and cultural adaptation to testing feasibility, acceptability, and preliminary efficacy through randomized controlled trials.^[71–73]^

The research directions of authors with high centrality focus on the development and application of technology-mediated JITAIs to reduce harmful substance use, with particular attention to the real-time prediction and intervention of lapse risks for behaviors such as smoking and drinking through EMA and sensor data.^[3,31,74]^Simultaneously, they are committed to utilizing novel experimental designs like MRTs to optimize intervention decision rules, assess proximal intervention effects, and address methodological challenges in digital intervention development.^[30,41,75]^Furthermore, their research also explores patterns and influencing factors of user engagement in digital behavior change interventions, as well as the causal mechanisms for enhancing engagement through notification strategies.^[76,77]^It also encompasses the co-design of JITAIs and the development of personalized intervention strategies for specific issues such as post-traumatic anger, stress management, and the promotion of walking behavior.^[28,78,79]^

### 4.5. Research hotspots

Integrating keyword cluster analysis and distribution characteristics, it is evident that research on JITAIs in the mental health field focuses on exploring the application of immediacy, adaptability, and the integration of digital technologies. The main research directions can be summarized into the following 3 aspects. First, technological and methodological innovation centered on “Just-in-Time Adaptive Intervention,” encompassing key technologies such as “Ecological Momentary Assessment,” “Mobile Health,” and “Machine Learning.” This drives the development of personalized, real-time intervention strategies, forming significant clusters particularly in areas like “smoking cessation,” “substance use disorders,” and “diet-related psychological issues.” Second, the expansion of clinical applications around themes like “mental health,” “emotion regulation,” and “behavioral adaptation,” addressing specific psychological problems such as “eating disorders,” “depression,” and “stress management.” These are implemented through carriers like “digital mental health,” “telemedicine,” and “mobile applications.” Third, differentiated intervention research targeting specific populations and contexts, such as “college students,” “low-income groups,” and “patients with chronic diseases.” This research focuses on issues like “health equity,” “social determinants,” and “cultural adaptation,” reflecting the field’s trend toward diversity and inclusivity in advancing precision mental health services. These 3 directions are derived from the keyword co-occurrence network (Fig. 6) and the 14 identified clusters (Fig. 7), highlighting the central roles of technological methods (e.g., EMA and mHealth), clinical targets (e.g., mental health and smoking cessation), and specific populations (e.g., college students and low-income groups).

#### 4.5.1. Technological and methodological innovation centered on “JITAI”

Research under this theme focuses on technological and methodological innovation centered on JITAIs. Its development heavily relies on the integration of advanced experimental designs, algorithmic models, and sensing technologies. MRTs, as an innovative experimental design, are widely used to assess the immediate effects and contextual moderating roles of intervention components within JITAIs, providing a causal inference foundation for constructing adaptive decision rules.^[7,80,81]^ At the algorithmic level, reinforcement learning (RL) and ML play key roles. The former is used to optimize personalized intervention strategies by continuously learning from user data to dynamically adjust the type, timing, and frequency of interventions.^[22,82,83]^ The latter is applied to predict high-risk moments (e.g., for binge eating, smoking, and drinking) by analyzing EMA and passive sensing data to achieve individualized risk identification.^[68,84,85]^ Meanwhile, advancements in sensor technology and passive monitoring enable the real-time capture of physiological, behavioral, and environmental data through wearable devices, providing JITAIs with real-time, objective “tailoring variables.”^[41,63,86]^ Furthermore, progress has been made in digital architecture and algorithm integration, such as scalable frameworks supporting the configuration of intervention logic based on rules or theories, and the incorporation of large language models and geographic information systems to generate personalized, context-aware intervention content.^[87–89]^ The fusion of these technological methods propels the evolution of JITAIs from simple notifications based on static rules towards sophisticated intervention systems that are highly personalized, context-aware, and driven by dynamic prediction, multisource data fusion, and artificial intelligence.^[19,27,47]^

#### 4.5.2. Expansion of clinical applications centered on themes of “mental health,” “emotion regulation,” and “behavioral adaptation”

The expansion of clinical applications around the themes of “mental health,” “emotion regulation,” and “behavioral adaptation” is significantly driven by technological and methodological innovations in JITAIs. These innovations are primarily demonstrated through their ability to achieve highly personalized, context-aware intervention delivery via multimodal sensing and real-time algorithmic decision-making.^[90,91]^ In the realm of mental health, systems based on EMA and smartphone sensors can continuously monitor emotional states (such as stress and anxiety) and contextual factors. They employ ML models to predict the risk of emotional deterioration, thereby triggering timely and appropriate supportive content or skill training.^[92,93]^ For instance, intervention systems for substance use disorders integrate heart rate variability monitoring from wearable devices with EMA-reported craving levels to identify high-risk moments and instantly deliver regulation exercises based on CBT or mindful breathing.^[94,95]^ In terms of behavioral adaptation, JITAIs analyze real-time data from global positioning system location, accelerometers, and self-report logs to precisely identify spatiotemporal and social contextual cues associated with high-risk behaviors (e.g., smoking and drinking).^[64,96]^ Subsequently, the system can automatically push customized coping strategies, such as suggestions for alternative activities for planned drinking or distraction techniques for smoking urges.^[97,98]^ Furthermore, the evolution of technological methods includes developing “digital phenotypes” to passively detect changes in behavioral patterns,^[99]^ and utilizing RL algorithms to dynamically optimize intervention type, dosage, and timing to maximize user engagement and intervention effectiveness.^[97,100]^ This integration of technologies transforms interventions from being static or predefined into dynamic adaptive systems capable of continuously learning individual behavioral patterns, psychophysiological responses, and environmental interactions. This provides a viable pathway for the precise and scalable prevention and treatment of issues such as anxiety,^[66]^ depression,^[101]^ addiction,^[102]^ and stress management.^[103]^

#### 4.5.3. Research on differentiated interventions for specific populations and contexts

Research on differentiated interventions for specific populations and contexts is a core manifestation of the expansion of JITAI clinical applications and technological innovation. Studies in this area have broadly encompassed diverse groups, including substance abusers (e.g., alcohol, tobacco, and opioid users)^[3,74,104]^; patients with mental illnesses (e.g., depression, anxiety, and post-traumatic stress disorder, and individuals at risk of suicide)^[6,105,106]^; and patients with chronic diseases (e.g., diabetes, cardiovascular disease, and obesity).^[46,81,107]^ To address the unique risk factors and behavioral patterns of different populations, research integrates mobile sensing data (e.g., global positioning system, accelerometer, and heart rate variability),^[104,108,109]^ EMA, and passive monitoring data to construct personalized risk prediction models. This enables precise adaptation of intervention content, timing, and intensity.^[8,47]^ Technological innovations have significantly advanced the practice of such differentiated interventions. For example, MRT designs are used to optimize intervention decision rules,^[18,30]^ ML and RL algorithms are applied to generate dynamic, adaptive intervention strategies,^[22,83,110]^ and natural language processing and generative artificial intelligence are leveraged to enhance interactivity and content personalization.^[99,111]^ These technologies enable interventions to not only respond to rapidly changing internal states (e.g., emotion, stress, craving),^[112,113]^ but also adapt to specific external contexts (e.g., geographic location, social environment, time points).^[114,115]^ Research focusing on predicting aggressive behavior in youth with autism spectrum disorder,^[55]^ intervening in substance use among individuals experiencing homelessness,^[65,116]^ and designing human immunodeficiency virus risk reduction measures for men who have sex with men^[117,118]^ further highlights the immense potential and clinical scalability of JITAIs in addressing complex public health problems and serving marginalized populations.

### 4.6. Research frontiers

Currently, the research frontiers in this field are closely centered on the application of JITAIs in mental health. The core trends are reflected in the refinement of research methodologies, the enhancement of technical implementation reliability, and the ecological orientation of intervention models. Specifically, research focuses on utilizing ecological momentary interventions to construct real-time, contextualized support systems,^[64,119,120]^ This method uses smartphones to deliver “just-right,” personalized therapeutic content within the user’s daily living environment. It is employed for managing substance use (e.g., alcohol, opioids, and tobacco) cravings and relapse prevention,^[94,119,121]^ as well as for improving mood disorders.^[120]^ To scientifically evaluate the momentary causal effects of individual intervention components within such dynamic interventions, MRTs have become a key research design paradigm,^[90]^ MRTs allow for randomization at different time points within an individual, enabling the testing of optimization strategies for intervention timing, content, or context, thereby providing an empirical basis for constructing adaptive intervention protocols.^[90,122]^

Simultaneously, ensuring the reliability of data derived from mobile sensing and self-reports is fundamental for propelling this field towards real-world application. Research frontiers include developing high-precision, passive detection algorithms for smoking behavior that are resistant to confounding gestures,^[123,124]^] validating the stability and accuracy of biomarkers from wearable devices for detecting psychological states like stress and craving,^[125]^ and examining the reliability of psychometric tools used in EMA,^[120]^ hese concerted efforts aim to enhance the reliability of predictions and feedback from just-in-time intervention systems, providing solid data support for effective “just-in-time” interventions. In summary, the 3 intertwined frontier directions of EMI, MRTs, and reliability validation are collectively driving JITAIs in mental health towards greater precision, robustness, and alignment with real-life contexts.

## 5. Conclusion

JITAIs, as personalized psychological intervention tools integrating mobile health, EMA, and artificial intelligence algorithms, have developed rapidly in the field of mental health in recent years. This bibliometric analysis based on 451 articles shows that, according to WOS data, the field has shown a marked increase in publications since 2021, which may indicate rapid growth. The United States holds a central position in both scientific output and international collaboration, with institutions such as the University of Michigan and the University of California system serving as the primary research forces.

Current research revolves around 3 major directions. First, methodological innovations represented by MRTs and RL, driving intervention strategies towards dynamic and personalized evolution. Second, clinical applications targeting specific psychological behaviors such as substance use, emotional dysregulation, and stress management, relying on mobile sensing and real-time algorithms to achieve precise intervention. Third, differentiated interventions focusing on marginalized groups, specific disorders, and cultural contexts to enhance the accessibility and equity of mental health services.

In summary, among the 451 articles analyzed, <10 reported follow-up data beyond the intervention period (data not shown in tables). This gap suggests that long-term effectiveness remains understudied and should be prioritized in future research. The bibliometric evidence, particularly the burst keywords (e.g., “validation” and “efficacy”), indicates that the field is currently prioritizing the validation of ecological momentary interventions and the assurance of data reliability. These are essential steps for transitioning JITAIs from experimental research toward practical application. Broader claims regarding scalability and ethical guidelines, while important, extend beyond the direct scope of the present data and should be interpreted with caution.

## 6. Limitations

This study has several methodological limitations. First, data were retrieved exclusively from the WOS Core Collection, excluding databases such as PubMed and Scopus. While WOS was selected for its standardized citation data essential for reliable co-citation analysis in CiteSpace,^[126,127]^ this single-source strategy inevitably introduces coverage bias, as WOS underrepresents regional journals, non-English publications, and conference proceedings. Consequently, our analysis likely omits relevant studies from Asia, South America, and Africa, and may miss cutting-edge findings presented only at conferences, which partially explains the predominantly Euro-American landscape observed in our collaboration network. Future bibliometric studies should adopt multisource data fusion strategies to present a more complete global research panorama. Second, we limited our search to articles and reviews, excluding conference papers, meeting abstracts, and dissertations, potentially removing emerging work in this rapidly evolving field. Third, co-citation analysis is retrospective and may not capture the very latest research frontiers. Despite these limitations, the core findings regarding research hotspots, collaboration patterns, and evolutionary trends remain valid within the defined scope of the WOS Core Collection.

## Author Contributions

**Conceptualization:** Lu Luo, Congling Li.

**Data curation:** Lu Luo.

**Formal analysis:** Congling Li.

**Funding acquisition:** Pingfang Liu, Congling Li.

**Investigation:** Pingfang Liu.

**Methodology:** Pingfang Liu.

**Project administration:** Aimin Wang, Pan Su, Wenjun Xie.

**Resources:** Pan Su.

**Software:** Pan Su, Lichun Tang.

**Supervision:** Aimin Wang, Lichun Tang.

**Validation:** Jianhui Zhou, Lichun Tang.

**Visualization:** Jianhui Zhou.

**Writing – original draft:** Wenjun Xie.

**Writing – review & editing:** Wenjun Xie.
